# Living with parents, lifestyle pattern and common mental disorders in adolescents: a school-based study in Brazil

**DOI:** 10.1186/s12889-022-14241-2

**Published:** 2022-10-05

**Authors:** Lucia Helena Almeida Gratão, Milene Cristine Pessoa, Luana Lara Rocha, Thales Philipe Rodrigues da Silva, Eloar dos Santos Freitas, Tatiana Resende Prado Rangel de Oliveira, Cristiane de Freitas Cunha, Larissa Loures Mendes

**Affiliations:** 1 School of Medicine, Pediatrics Department, Universidade Federal de Minas Gerais, Belo Horizonte, Minas Gerais Brazil; 2 School of Nursing, Department of Nutrition, Universidade Federal de Minas Gerais, Belo Horizonte, Minas Gerais Brazil; 3 Medicine School, Departament of Preventive and Social Medicine, Universidade Federal de Minas Gerais, Belo Horizonte, Minas Gerais Brazil; 4 Nutrition Course, Pontifícia Universidade Católica de Minas Gerais, Belo Horizonte, Minas Gerais Brazil

**Keywords:** Mental Health, Lifestyle, Parents, Adolescent health

## Abstract

**Background:**

Mental health conditions represent 16% of the global burden of disease and injury in adolescents. Promotion, protection, and restoring the mental health must be considered indispensable, especially in adolescence. This study aims to verify the association of lifestyle pattern, living with parents and the presence of Common Mental Disorders (CMD) in Brazilian adolescents.

**Methods:**

Cross-sectional study that analyzed data from 71,553 adolescents aged 12–17 years, from the Study on Cardiovascular Risks in Adolescents (ERICA), between 2013 and 2014. Principal Component Analysis (PCA) was performed to identify lifestyle pattern, and Logistic Regression Models were performed to identify the associations between lifestyle pattern, living with parents, and presence of CMD.

**Results:**

To construct the Common Mental Disorders (CMD) variable, the Goldberg General Health Questionnaire was used. The Pattern of Healthy Lifestyle Practices found was characterized by higher water consumption, lower consumption of ultra-processed foods, the habit of eating breakfast, less exposure time to screens, habit of physical activity, and longer mean sleep time in hours. Adolescents belonging to the second (OR: 0.73; 95% CI 0.65–0.82) and third (OR: 0.44; 95% CI 0.39–0.50) terciles of the pattern, that is, those who had higher belonging to the pattern had lower chances of having CMD. Adolescents who lived with neither parent (OR: 1.44; 95% CI 1.16–1.78) were associated with a higher chance to present CMD.

**Conclusion:**

Living with parents can contribute to better mental health among adolescents. In addition, the adoption of a healthy lifestyle, encouraged by parents and the community, can reduce the chances of CMD in Brazilian adolescents.

## Background

Mental health can be defined as a state of well-being in which the individual, with their skills, can deal with everyday tensions, be productive and contribute to their community. [1] Therefore, promotion, protection, and restoring mental health must be considered indispensable to everyone, both individually and collectively, and in all age groups. [1, 2].

Special attention should be paid to adolescence, in which a significant percentage of these disorders start in childhood, adolescence or young adulthood, with more than half of the cases occurring around 14 years old. [3, 4] Worldwide, mental health conditions represent 16% of the global burden of disease and injury in adolescents. [2] In Brazil, data obtained from a representative study for the Brazilian adolescents, in 2014, found a 30% prevalence of Common Mental Disorders (CMD). [5] This information was produced in the same database (ERICA project) that is being re-analyzed in the current work, where are being proposing to carry out a new analysis to find other associations with CMD.

Some studies have shown associations between lifestyle habits, such as sleep, diet, physical activity, hydration, and sedentary behavior [6]. However, the published studies have isolated associations, without considering the adoption of multiple lifestyle habits. In Brazil there are no studies with national representativeness that have evaluated these associations. An important factor in the adoption of a healthy lifestyle is the presence of parents in the household. [7, 8].

Therefore, testing the hypothesis that the adoption of a healthy lifestyle is associated with the presence of CMD, being the parents mediating the adoption of this lifestyle, we conducted the present study, which aims to verify the association of lifestyle pattern, living with parents and the presence of CMD in Brazilian adolescents.

## Methods

### Design, sample, study participants and data collection

The data for this study was obtained from the Study on Cardiovascular Risks in Adolescents (Portuguese acronym, “ERICA”, Estudo de Riscos Cardiovasculares em Adolescentes). ERICA was a cross-sectional, national, school-based study with data collection carried out between March 2013 and December 2014, with a sample of adolescents aged 12 to 17 years old of both sexes, enrolled in 1,251 schools public and private schools, in 124 Brazilian municipalities. [9].

Three questionnaires were applied: for adolescents, for parents/educators, and about the school. [9] For the analysis of the present study, only the questionnaire for adolescents was used, including a 24-hour recall (R24h). So, the information from 71,553 adolescents were eligible used in the analyses.

Detailed information on the sampling process, research protocol, participant selection, and data collection can be found in studies previously published by the ERICA Study Committee. [9–11].

### Dependent variable

To construct the Common Mental Disorders (CMD) variable, the Goldberg General Health Questionnaire (GHQ-12)(1972) was used, validated for use in adolescents. [12] The GHQ-12 is a widely used self-administered instrument and is known to be a reliable measure of mental health. [13].

For the screening of CMD among adolescents, the binary system with a cutoff point of five was considered, that is, the presence of CMD was considered when at least 5 of the 12 items were answered with one of the last two options of the questionnaire (“a little more than normal” or “much more than normal”). This cut-off point has a sensitivity of 73.0%, specificity of 90.0%, a positive predictive value of 61.2%, and ROC curve area (Receiver Operating Characteristics) of 0.90. [14].

### Independent variables

#### The lifestyle pattern

For the construction of thelifestyle pattern, the following ERICA’s variables were used: daily water consumption, percentage of ultra-processed food consumption, breakfast consumption, hours of exposure to screens, physical activity, and mean sleep time in hours.

The variable water consumption, obtained from the question “How many glasses of water do you drink in a day?“, was categorized into “Consumption greater than five glasses of water a day” and “Consumption less than five glasses of water a day”. This categorization was performed according to the possible answers to this question in the ERICA Study database, which would be: “Do not drink water”, “Drink 1 to 2 glasses a day”, “Drink 3 to 4 glasses of water a day” and “Drinks at least 5 or more glasses of water a day”.

The percentage of consumption of ultra-processed food was calculated based on information from the R24h, applied through face-to-face interviews carried out by trained researchers. The interview technique used was the multiple-pass method, which consists of a guided interview in five stages, to reduce underreporting of food consumption. [15] The Brazil-Nutri software [16] was used to record food consumption data. The software used had a list of 1,626 food, from the database on the acquisition of food and beverages in the Family Budget Survey of 2002–2003, (Portuguese acronym, POF), carried out by the Brazilian Institute of Geography and Statistics (Portuguese acronym, IBGE)[17, 18]. The POF provides information on the household budget composition and on the living conditions of the population, including the subjective perception of quality of life, as well as generating databases and studies on the nutritional profile of the population[17, 18].

After converting the weight of the food items into grams, the dataset was linked to a nutritional composition Tables[19] to calculate the energy consumption of each adolescent. The foods were classified based on the degree of processing, as indicated by the NOVA food classification system [20]. This classification system categorizes all foods into the following 4 groups, according to the nature, extent, and purpose of the industrial processes they undergo: unprocessed and minimally processed food, processed culinary ingredients, processed food, and ultra-processed food. [20] The culinary preparations were disaggregated and their ingredients classified into their respective groups. The food was categorized by 2 independent researchers and discrepancies, if any, were resolved by an expert researcher.

The percentage of energy in Kcal from ultra-processed food concerning the total amount of energy ingested on the day evaluated. Outliers were excluded from the present study those. Were considered outliers participants who had a food intake below 500 Kcal/day or above 6,000 Kcal/day. [21] For the purposes of analysis in this study, this variable was not categorized, and its continuous numerical form was used.

The breakfast consumption variable was obtained from the question “Do you eat breakfast?”. The categories of the variable adopted were: “Does not have breakfast”, “Has a habit of consuming breakfast sometimes” and “Has a habit of consuming breakfast regularly”.

Screen time was investigated by the question “On a common weekday, how many hours do you use a computer or watch TV or play video games?“. The variable was categorized according to the recommendation of the Brazilian Society of Pediatrics, [22] as “≤ 3 hours a day in front of screens” and “> 3 hours a day in front of screens”.

The categorization of the time of weekly physical activity level practice was performed according to National Adolescent Health Survey (Portuguese acronym: PENSE) [23], in which adolescents who accumulated 300minutes or more of physical activity per week were considered physically “active”, “insufficiently active 1” those between 1 and 149minutes, “insufficiently active 2” those who practiced any Physical activity level from 150 to 299min. Students who did not practice any Physical activity level in the week before the interview were considered “inactive”. [23].

To obtain the variable mean sleep time, the weighted mean between the time in hours of sleep usually practiced during weekdays and weekend days was calculated, separately. Those individuals who reported sleeping less than 4hours and more than 14hours were not considered, for not meeting the usual parameters of sleep in this age group, according to Borges. [24] For analysis of this study, this variable was not categorized, and its continuous numerical form was used.

Principal Component Analysis (PCA) was used to calculate the lifestyle pattern. The Kaiser-Meyer-Olkin (KMO) was estimated as a measure of adequacy of the PCA, with values between 0.5 and 1.0 considered acceptable for this index. Subsequently, the components with eigenvalue greater than 1.0, defined according to the screen plot graph, were extracted from the PCA. The structure of the component was obtained by the indicators that presented factor loads greater than 0.3 or less than − 0.3.The results identified a main component, which was renamed as Pattern of Healthy Lifestyle Practices, are presented in Table1), with a contribution of 21.99% of explained accumulated variance. The KMO index and the factor loadings of all indicators were satisfactory. The pattern was characterized by higher water consumption, lower consumption of ultra-processed foods, the habit of eating breakfast, less exposure time to screens, habit of physical activity, and longer mean sleep time in hours.

**Table 1:** Factor loadings of the Pattern of Healthy Lifestyle Practices of Brazilian adolescents. ERICA, Brazil, 2013–2014

Indicators	Pattern of healthy living practices	KMO^a^
Water consumption	0.4388	0.5580
Percentage of consumption of ultra-processed food	-0.3854	0.6063
Habit of having breakfast	0.5068	0.5843
Daily screen hours^b^	-0.4255	0.5838
Mean sleep hours	0.3486	0.5504
Practice of physical activity	0.3155	0.5407
*Eigenvalue*	*1.31911*	.
*Explained variance (%)*	*21.99*	.
*Overall*	.	*0.5696*

^a^Kaiser-Meyer-Olkin

^b^ Only televisions, computers and video games were considered as devices.

#### Living with parents

Considering that daily interactions with parents during adolescence can be important for the development of healthy lifestyle, the variable “living with parents” from the ERICA study was categorized into two categories: “lives with both parents or live only with mother or only father” and “does not live with parent”. The original variable had all three categories individually.

### Adjustment variables

**Fig. 1:**
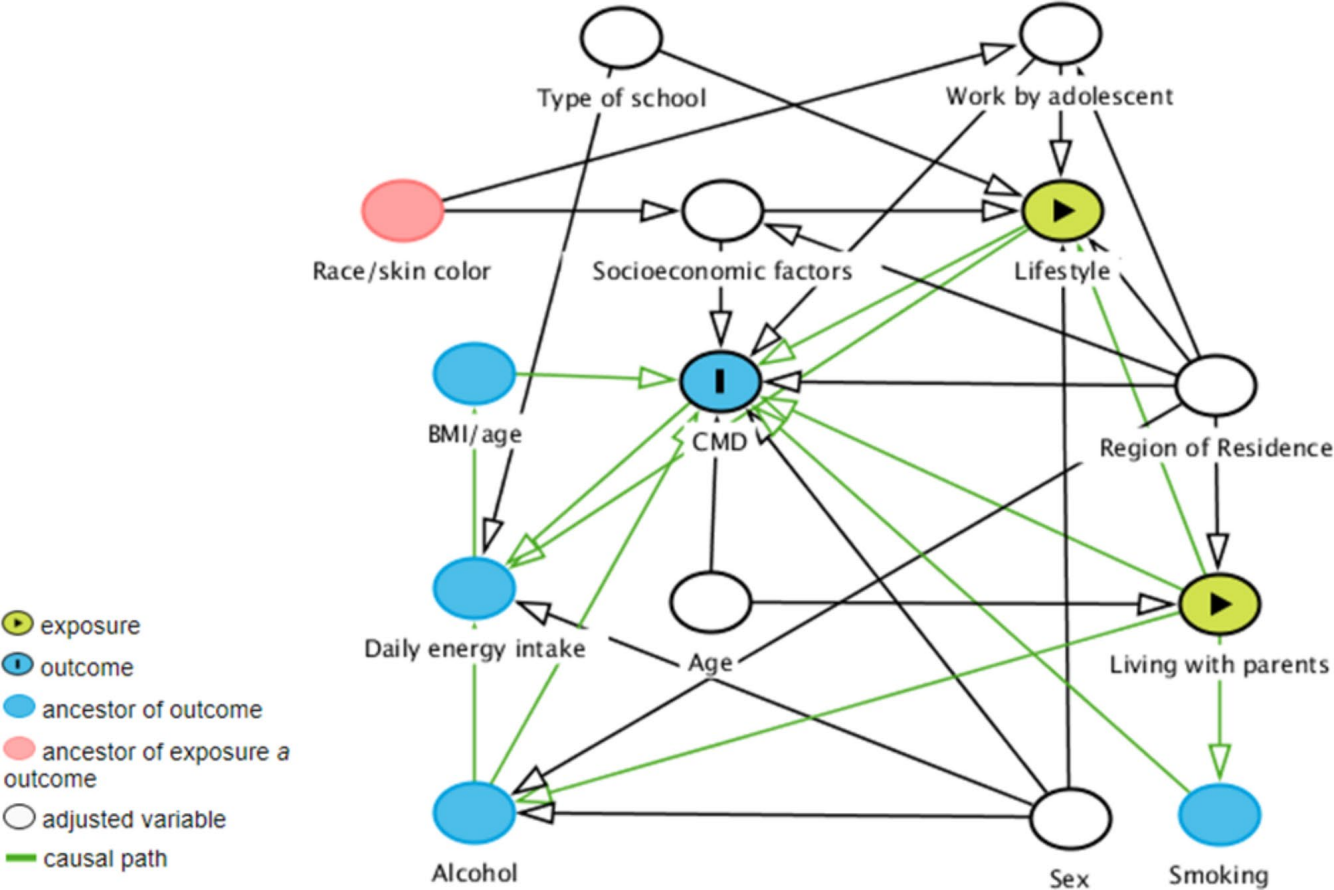
Directed Acyclic Graph: lifestyle and living with parents (exposure) and Common Mental Disorders (CMD) (outcome)

The adjusted variables were identified from a theoretical model and selected with the aid of a Directed Acyclic Graph (DAG) built in the DAGitty [25]. The model was carried out considering the outcome variable CMD and the explanatory variables lifestyle and living with parents (Fig.1).

The set of minimum adjustments sufficient to estimate the total effect of a healthy lifestyle and living with parents with CMD recommended by the DAG were: age, region of residence, sex, socioeconomic factors, type of school, work by adolescent (Fig.1).

The age of the adolescents was categorized into three age groups: 12 and 13, 14 and 15, 16 and 17. As for sex, the alternatives in the student’s questionnaire were: female and male.

The variable region of residence identifies the five Brazilian regions: North, South, Midwest, Northeast, and Southeast.Type of school could be public or private administration.

The variable work by adolescent, was constructed from two variables from the questions “Has the student worked without pay in the last year?” and “Student worked with pay in the last year?”, that is, the performance of paid or/and unpaid activities was considered as work. Therefore, the categories of the variable considered for the model were “No” and “Yes”.

To better characterize the socioeconomic pattern of the adolescents’ families, a pattern of socioeconomic indicators was calculated from proxy variables, described by Ribeiro et al. [26] and Erwling and Barros [27]. For this was performed the Principal Component Analysis (PCA). The proxy variables considered were: “number of residents per room”, “employees in the residence”, “number of bathrooms” and “number of refrigerators”.

The Kaiser-Meyer-Olkin (KMO) was estimated as a measure of adequacy of the PCA, with values between 0.5 and 1.0 considered acceptable for this index. Subsequently, the components with eigenvalue greater than 1.0, defined according to the screen plot graph, were extracted from the PCA. The structure of the component was obtained by the indicators that presented factor loads greater than 0.3 or less than − 0.3, being generated a variable in score units for the socioeconomic patterns, named as Pattern of Socioeconomic Indicators. A categorical variable was created from the tercile values of the distribution of scores of these patterns.

The Pattern of Socioeconomic Indicators is characterized by the presence of employees, lower number of residents per room, higher number of bathrooms and higher number of refrigerators in the residence (Table2).

**Table 2:** Factor loads of the first component of the Principal Component Analysis of the Pattern of Socioeconomic Indicators of Brazilian adolescents included in the ERICA study. Brazil, 2013–2014

Indicators	Pattern of Socioeconomic Indicators	KMO^¥^	
Presence of employees	0.4668	0.6162	
Number of residents per room	-0.4315	0.5213	
Number of bathrooms	0.6417	0.5365	
Number of refrigerators	0.4291	0.5601	
*Eigenvalue*	*1.44892*	.	
*Explained variance (%)*	*36.22*	.	
*Overall*	.	*0.5505*	

^¥^Kaiser-Meyer-Olkin

### Statistical analysis

Descriptive analysis included the calculation of absolute and relative frequencies for categorical variables, in addition to measures of central tendency. The chi-square test was performed to compare proportions between variables.

Crude analysis was performed using a multiple logistic regression model, considering the presence of CMD as a dependent variable, and Pattern of Healthy Lifestyle Practices and living with parents as independent variables. In the adjusted analysis the variables identified in the causal diagram were included: age, region of residence, sex, Pattern of Socioeconomic Indicators, type of school, paid or unpaid work by adolescent.

The ERICA sample is considered a complex sample, since it employs stratification and conglomeration and unequal probabilities in its selection stages [11]. It is noteworthy, that because the data from the ERICA Study come from a complex sample, the survey command (svy:) was applied in all statistical analyses, which were performed in the Stata 14.0 software, considering the expansion factors. The odds ratio (OR) with a 95% confidence interval (95%CI) was used as a measure of effect.

### Ethical aspects

This report was approved by the Research Ethics Committee of the Instituto de Estudos de Saúde Coletiva da Universidade Federal do Rio de Janeiro (IESC/UFRJ) which belongs to the report’s central coordination (IESC/UFRJ – Aprovation nº 45/2008) and of each Brazilian State (Rio Grande do Sul, Santa Catarina, Paraná, São Paulo, Rio de Janeiro, Espírito Santo, Minas Gerais, Bahia, Goiás, Mato Grosso do Sul, Distrito federal, Tocantins, Mato Grosso, Rondônia, Acre, Amazonas, Pará, Amapá, Roraima, Maranhão, Ceará, Rio Grande do Norte, Paraíba, Pernambuco, Alagoas, Sergipe e Piauí. Informed consents were obtained from all subjects, parent and their legal guardian(s). The authors confirm that all methods were performed in accordance with the Declaration of Helsinki.[28]

## Results

### Sample characteristics

In this study, data from 71,553 Brazilian adolescents were evaluated. This amostral number was obtained among the adolescents who answered the R24h and completed the student questionnaire. The prevalence of CMD in these adolescents was 17.10% (cut-off point 5 for the GHQ-12).

Table3 shows the characterization of the adolescents, it was observed that the presence of CMD was more prevalent among girls adolescents (23.30%), aged between 16 and 17 years (20.30%), who perform work activities (20.13%), who does not live with any of the parents (23.23%), and belong to the first tertile of the Pattern of Healthy Lifestyle Practices , that is, those who had less healthy lifestyle practices (22.81%).

**Table 3:** Characterization of Brazilian adolescents with presence of Common Mental Disorders. ERICA, Brazil 2013–2014, (n = 71,553)

Variable	Total Sample (n)^¥^	Total Sample (%)^§^	CMD- (%)^§^	CMD+ (%)^§^	*p-*value^a^
**Sex**					
Female	39,690	49.79	76.70	23.30	**< 0.001**
Male	31,863	50.21	89.90	10.91	
**Age (Years)**					
12–13	19,883	35.10	86.19	13.81	**< 0.001**
14–15	26,670	34.99	82.40	17.60	
16–17	25,050	29.90	79.70	20.30	
**Pattern of Socioeconomic Indicators** ^**b**^					0.284
Tertile 1	31,609	46.26	82.68	17.32	
Tertile 2	24,864	35.04	83.66	16.34	
Tertile 3	14,349	18.70	82.53	17.47	
**Paid or unpaid work by adolescent**					
No	54,190	73.97	83.99	16.01	**< 0.001**
Yes	17,363	26.03	79.87	20.13	
**Region of residence**					
Middle West	9,331	7.67	82.23	17.77	0.791
Northeast	22,205	21.34	83.21	16.79	
North	14,494	8.43	82.44	17.54	
South East	16,434	50.78	83.06	16.94	
South	9,089	11.78	82.59	17.41	
**Living with parents**					**< 0.001**
Both or with mother/ father	66,809	94.12	83.30	16.70	
Neither parents	4,919	5.88	76.77	23.23	
**Type of school**					0.403
Public	56,703	83.61	83.01	16.99	
Private	14,850	16.39	82.47	17.53	
**Pattern of Healthy Lifestyle Practices** ^**c**^					
Tertile 1	18,914	34.08	77.19	22.81	**< 0.001**
Tertile 2	18,914	32.78	83.42	16.58	
Tertile 3	18,912	33.15	90.02	9.98	

^a^ The chi-square test

^b^ The pattern of socioeconomic indicators was characterized by a higher number of employees in the home, a lower number of residents per room, a higher number of bathrooms in the home and a higher number of refrigerators in the home (Table1)

^c^The pattern of healthy habits was characterized by consumption of water more than 5 glases/day, less percentage of consumption of ultra-processed foods, habit of eating breakfast, time of exposure to screens less or equal 3h/day, habit of practice physical activity and longer mean sleep time in hours (Table3)

**Boldface** indicates statistical significance (p < 0.05).

^¥^ Sample number without using sample weight.

^§^ Frequency of the sample using sample weight, extrapolable to the Brazilian population.

### Association between Pattern of Healthy Lifestyle Practices, living with parents and Common Mental Disorders

The variables associated with the presence of CMD in Brazilian adolescents in the logistic regression model are shown in Table4. Based on the results, we identified that adolescents belonging to the first tercile (OR: 1.36; 95%CI 1.21–1.52), that is, those who had less healthy lifestyle practices had more chance of CMD+. Moreover, those who belonged to the third tercile (OR: 0.61; 95%CI 0.53–0.70), that is, those who have a healthier pattern of lifestyle practices, had lower chances of CMD+. Moreover, adolescents who lived without their parents (OR: 1.44; 95%CI 1.16–1.78) were associated with a higher chance to present the outcome (Table4).

**Table 4:** Crude and adjusted logistic regression analysis. ERICA Brazil, 2013–2014, (n = 71,553)

Variable	Common Mental Disorders	
	**Crude** **OR (CI 95%)**	**Adjusted** **OR (CI 95%)** ^**a,¥**^
**Pattern of HealthyLlifestyle Practices** ^**b**^		
First tercile	1.49 (1.33–1.66)***	1.36 (1.21–1.52)***
Second tercile	(Ref.)	(Ref.)
Third tercile	0.56 (0.49–0.63)***	0.61 (0.53–0.70)***
**Living with parents**		
With both or only mother/father	(Ref.)	(Ref.)
Neither parent	1.51 (1.26–1.82)***	1.44 (1.16–1.78)***

OR: Odds Ratio; CI: Confidence Interval

^a^ Adjusted by age, region of residence, sex, socioeconomic factors, type of school, work by adolescent.

^b^ The pattern of healthy habits was characterized by consumption of water more than 5 glases/day, less percentage of consumption of ultra-processed foods, habit of eating breakfast, time of exposure to screens less or equal 3h/day, habit of practice physical activity and longer mean sleep time in hours (Table3).

^¥^ Goodness of fit of final model: p = 0.4142

* Indicates statistical significance (*p < 0.05, **p < 0.01, ***p < 0.001).

## Discussion

Adolescents live in environments that expose them to multiple risk and protective factors simultaneously, promoting the interaction of these factors that can influence the mental health. So, the proposal of this study was to attempt a pattern, to better understand the aggregation of multiple lifestyle practices and their association with the presence of CMD in adolescents. Our results showed an inverse association between belonging to the healthy lifestyle pattern and the presence of CMD, and a direct association between lived without their parents and CMD in Brazilian adolescents.

The adoption of multiple protective factors, adopting a healthy lifestyle, considering good diet, hydration, physical activity, less time spent in sedentary activities, can be effective in preventing CMD [7, 29]. Loewen et al. [7], in a prospective study with groups of Canadian adolescents, found that those who adopted 4 to 6 health recommendations of lifestyle, compared to those who followed only 1 to 3, had 39% less visits to the specialist in mental health. Those who followed 7 to 9 recommendations had 56% less visits over the three years of the study, reinforcing the importance of adhering to multiple recommendations for the prevention of mental disorders.

Adolescence is a crucial period for the development of personality, self-esteem, and lifestyle, being a period of opportunity to promote protective habits for mental health, and the family plays an essential role in this context. [30] Minuzzi et al. (2019), [31] verify the association of parents’ lifestyle profiles with those of their children, found that positive parental behavior, that is, behaviors that promote health and quality of life, increases the chances of positive behavior by the children. In addition, some studies have shown that the family and the parenting styles adopted, especially authoritarian and neglectful styles, can contribute to the worsening of mental health in adolescence, as the form of treatment, attitudes, and quality of care relationships with parents influence socio-emotional development. [32–34].

In the present study, who lived without their parents was associated with increased odds for the presence of CMD. By living with their parents, adolescents may have greater chances of contacting those responsible, strengthening the parent-child bond. The strengthening of this bond and attachment to parents is inversely associated with emotional difficulties, fewer conduct problems, and prosocial behavior. [35] It is observed that the active presence and support of parents or responsible, can contribute to better mental health outcomes in adolescents. [35].

It is important to note, that the absence of parents, the explanatory variable analyzed in this study, is a non-modifiable condition, different from the healthy lifestyle pattern. Therefore, this characteristic should be investigated and used by health services and professionals to recognize groups of adolescents more vulnerable to have CMD+.

Our study has some limitations, as the cross-sectional design, without the possibility of causal inferences and with the possibility of reverse associations. Finally, the reducing of variables (PCA analysis) is useful in the analyses, but not for decision makers. The use of the results from this variable does not facilitate the direct identification of the more vulnerable groups.

This study contributes to the field of adolescent mental health studies, demonstrating that the adoption of a healthy lifestyle, already widely recommended for chronic non-communicable diseases as form of prevention and treatment, can also be oriented towards the prevention of CMD in the population studied.

## Conclusion

This study identified an inverse association between a Pattern of Hhealthy Lifestyle Practices and the presence of CMD, and a direct association between not living with parents and the presence of CMD in Brazilian adolescents. The findings reinforce that the orientation of practices already consolidated for other diseases, such as the adoption of a healthy lifestyle is important for the mental health of adolescents. It is important to emphasize that the success of this adoption requires the involvement of the adolescent, the family, the community, and the government, in order to guarantee structure and support for the consolidation of the practices.

## Data Availability

The datasets used and/or analysed during the current study are available from the ERICA Study Comitee on reasonable request.

## Abbreviations

CMD: Common Mental disorders.
ERICA: Report on Cardiovascular Risk in Adolescents.
GHQ-12: Goldberg General Health Questionnaire.
PCA: Principal Component Analysis.
ROC: Receiver Operating Characteristics.
IBGE: Instituto Brasileiro de Geografia e Estatística.
DAG: Directed Acyclic Graph.
WHO: World Health Organization.
KMO: Kaiser Meyer Olkin.
FCP: Food Consumption Patterns.
R24h: 24-hour recall
